# The Influence of Articular Cartilage Thickness Reduction on Meniscus Biomechanics

**DOI:** 10.1371/journal.pone.0167733

**Published:** 2016-12-09

**Authors:** Piotr Łuczkiewicz, Karol Daszkiewicz, Jacek Chróścielewski, Wojciech Witkowski, Pawel J. Winklewski

**Affiliations:** 1 II Clinic of Orthopaedics and Kinetic Organ Traumatology, Medical University of Gdańsk, Gdańsk, Poland; 2 Department of Mechanics of Materials, Faculty of Civil and Environmental Engineering, Gdańsk University of Technology, Gdańsk, Poland; 3 Institute of Human Physiology, Medical University of Gdańsk, Gdańsk, Poland; 4 Institute of Health Sciences, Pomeranian University of Słupsk, Słupsk, Poland; University of Umeå, SWEDEN

## Abstract

**Objective:**

Evaluation of the biomechanical interaction between meniscus and cartilage in medial compartment knee osteoarthritis.

**Methods:**

The finite element method was used to simulate knee joint contact mechanics. Three knee models were created on the basis of knee geometry from the Open Knee project. We reduced the thickness of medial cartilages in the intact knee model by approximately 50% to obtain a medial knee osteoarthritis (OA) model. Two variants of medial knee OA model with congruent and incongruent contact surfaces were analysed to investigate the influence of congruency. A nonlinear static analysis for one compressive load case was performed. The focus of the study was the influence of cartilage degeneration on meniscal extrusion and the values of the contact forces and contact areas.

**Results:**

In the model with incongruent contact surfaces, we observed maximal compressive stress on the tibial plateau. In this model, the value of medial meniscus external shift was 95.3% greater, while the contact area between the tibial cartilage and medial meniscus was 50% lower than in the congruent contact surfaces model. After the non-uniform reduction of cartilage thickness, the medial meniscus carried only 48.4% of load in the medial compartment in comparison to 71.2% in the healthy knee model.

**Conclusions:**

We have shown that the change in articular cartilage geometry may significantly reduce the role of meniscus in load transmission and the contact area between the meniscus and cartilage. Additionally, medial knee OA may increase the risk of meniscal extrusion in the medial compartment of the knee joint.

## Introduction

Menisci are fibrocartilaginous structures which play a very important role in the mechanical protection of knee cartilage [1,2]. They distribute stress, absorb shocks, enhance congruity and stabilise the knee joint. The axial load from the body weight is transferred by femoral condyles to the menisci, yielding their motion in the radial direction. Thanks to the predominant circumferential orientation of the collagen fibres in the menisci, they counteract extrusion and direct tensile forces along their axis. For vertical compression they carry between 44% and 78% of the load, reducing the mechanical stresses on cartilage surfaces [3]. It is well accepted that the loss of meniscal integrity may reduce radial stiffness and cause radial displacement, which is significantly associated with increased peak tibiofemoral contact stress and cartilage degeneration [4,5].

Although meniscal pathology occurs in 63% of adults with symptomatic knee osteoarthritis (OA) and precedes other pathological conditions, its role in disease progression is still unclear [6,7,8,9,10]. It remains unclear why the intact meniscus is not able to decrease tibiofemoral contact stresses in medial knee osteoarthritis, why it is paradoxically hypertrophied in varus knee OA or why intact menisci alter their position in medial compartment knee OA [11,12,13]. The previous studies did not sufficiently explain the differences in meniscal biomechanics, leaving the analysis of pathology under speculation. Despite the multifactorial nature of this disease, the pathological changes seen in knee OA affect the entire joint and include hyaline cartilage damage, inflammation of the synovium, degeneration of the ligament and menisci, hypertrophy of the joint capsule, thickening of the subchondral bone and the formation of osteophytes. The observation of interaction between tissues is needed to evaluate the risk of disease progression and to determine the pathomechanics of the disease [9]. Since mechanical abnormalities are predominant among other knee OA factors, the mechanical relationship between meniscus and cartilage in the development of osteoarthritis is very important [14].

The goal of this study is to examine the influence of cartilage thickness on the ability of the menisci to distribute load. To investigate this issue, we carried out finite element analysis (FEA) on a knee model with reduced thickness of the medial compartment cartilages. FEA helps to explain the individual role of tissues in knee contact mechanics. The scientific novelty of the paper is evaluation of the influence of articular cartilage degeneration on meniscus extrusion in both knee joint compartments. Moreover, we investigated the distribution of stresses in the knee joint after the articular cartilage thickness reduction. Using the FEA, it is possible to assess the effect of only one chosen factor, unlike in experimental tests, where this is difficult because of the many coexisting pathological changes seen in the OA knee.

## Method

### Geometry

**Fig 1:**
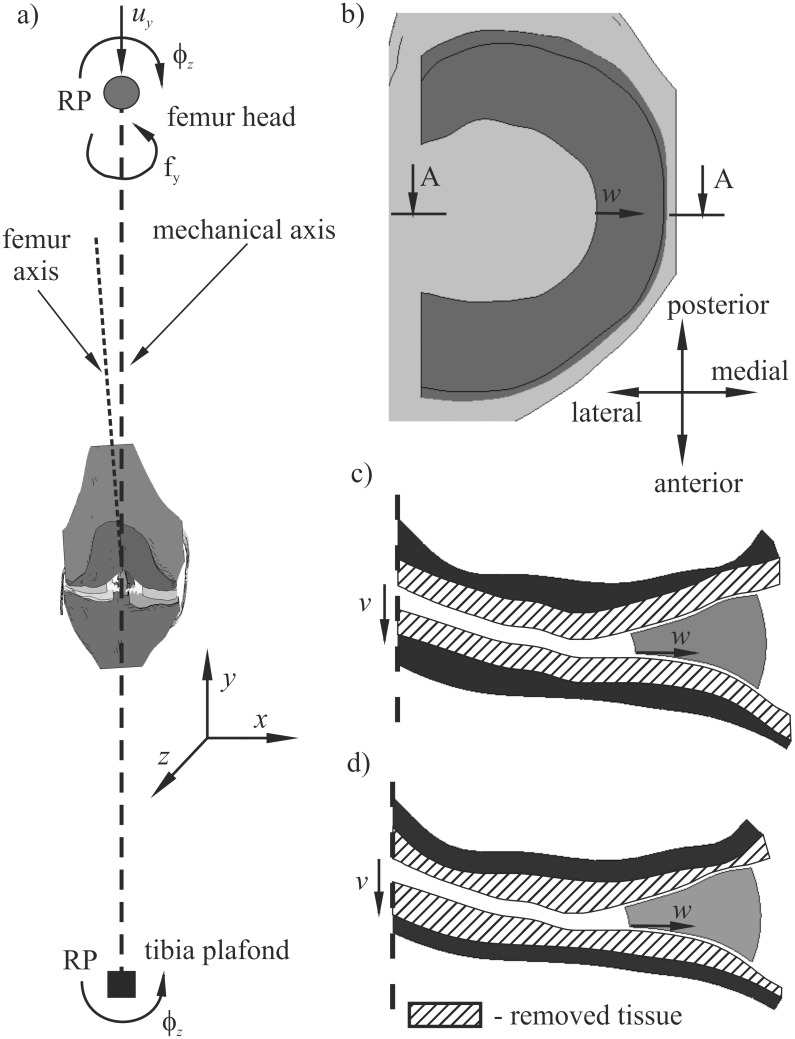
The geometry of the knee models, a) coronal view, free degrees of freedom at reference points (RP); b) the position of A-A cross section in axial view; c) cross section A-A of medial meniscus and articular cartilages for model 2 with congruent contact surfaces; d) cross section A-A of medial meniscus and articular cartilages for model 3 with incongruent contact surfaces; *w*–meniscus external shift, *v*–vertical shift of the articular surfaces.

Three finite element models were created using a freely accessible and customisable geometry of knee joint from the Open Knee project [15,16]. The geometry was prepared on the basis of the right cadaver knee (female donor, 1.68 m, 77.1 kg, 70-year old) [17]. Bones (tibia, femur), articular cartilages, menisci and ligaments: anterior cruciate ligament (ACL), posterior cruciate ligament (PCL), lateral collateral ligament (LCL), and medial collateral ligament (MCL) were taken into account in the knee finite element method (FEM) model (Fig 1a). The kinematics of the rigid bones were defined by the so-called reference points (RPs) placed on the mechanical axis of the knee (Fig 1a). These points were assumed in the centre of the femur head and the centre of the tibia plafond to properly simulate the shift of mechanical axis during varus/valgus rotation. The position of the RPs was calculated on the basis of bone length, which was estimated with respect to donor height and statistical data [18].

In the basic model, referred to as model 1, the geometry from the Open Knee project was used without any changes. Degeneration of cartilage in the medial compartment during the osteoarthritis process was simulated by decreasing their thickness by around 50%. Two variants of this model were introduced: model 2 and model 3, with congruent and incongruent articular surfaces, respectively. In model 2, the thickness of the tibial and femoral medial cartilages was reduced uniformly to obtain surface profiles as in the original model (Fig 1c). In model 3, constant thickness of the degenerated articular cartilages was obtained by non-uniform (site-specific) reduction of their volume (Fig 1d), according to the literature data for “moderate” stage OA [11].

In the analyses, we measured the displacements specified in this section. The displacement *w* of the medial and lateral meniscus was calculated on the internal periphery in the middle part of the meniscus (Fig 1) in the medial and lateral direction, respectively. The external shift of meniscus *w* was computed relative to the tibia plateau to show motion between the meniscus and the bone. The relative vertical displacements *v* (Fig 1c and 1d) between the articular cartilages were measured to give information about joint space narrowing. The values of *v* were computed in central points of contact areas between tibial and femoral cartilages, separately for both compartments. We also measured a relative displacement *u* between articular surfaces of bones in the lateral-medial direction. The value of *u* was calculated in the centre of the knee joint as the difference between the displacement in the medial direction of the femur and the tibia. The varus rotation for the knee joint was computed as the sum of tibia and femur rotations in the coronal plane.

### Materials

In the FEM analyses, the material used for articular cartilage was linear, elastic and isotropic, with an elastic modulus *E* = 10 MPa and a Poisson ratio of *v* = 0.45 [19,20]. The assumed material properties for transversely isotropic material model of menisci are presented in Table 1. The bones were defined as rigid shells, because of their large stiffness in comparison to soft tissues. Young’s moduli of meniscal horn attachments: 161 MPa for lateral anterior, 96.3 MPa for lateral posterior, 179 MPa for medial anterior and 85.3 MPa for medial posterior were taken from the paper [21]. The attachments were modelled by linear springs connecting nodes on meniscal horn faces with insertion nodes on tibial surface. The spring constants for the meniscal attachments were computed in the paper [22].

**Table 1:** Material parameters for menisci and ligaments.

	*E*_*θ*_ [MPa]	*E*_*r*_,*E*_*z*_ [MPa]	*v*_*rθ*_,*v*_*zθ*_[-]	*v*_*rz*_ [-]	*G*_*rθ*_,*G*_*zθ*_ [MPa]	*G*_*rz*_ [MPa]	Source
menisci	120	20	0.3	0.2	57.7	8.33	[23]
	*C*_1_ [MPa]	*D*_1_ [MPa^-1^]	*C*_3_ [MPa]	*C*_4_ [-]	*C*_5_ [MPa]	λ* [-]	Source
ACL	1.95	0.01366	0.0139	116.22	535.039	1.046	[24]
LCL	1.44	0.00252	0.57	48.0	467.1	1.062	[25]
MCL	1.44	0.00252	0.57	48.0	467.1	1.062	[25]
PCL	3.25	0.0082	0.1196	87.178	431.063	1.035	[24]

*E*–Young’s modulus, *v*–Poisson ratio, *G*–shear modulus (subscripts *θ*, *r*, *z* refer to the constants in the circumferential, radial and axial direction, respectively); *C*_1_, *D*_1_ –Neo-Hookean constants; *C*_3_, *C*_4_, *C*_5_ –parameters describing reinforcement, λ*–limit value of stretch.

Nearly incompressible, hyperelastic, transversely isotropic material model was used to describe the behaviour of ligaments. The strain-energy function

$$\Phi =C_{1}({\bar{I}}_{1}-3)+\frac{1}{D_{1}}{\left(J-1\right)}^{2}+F_{2}(\lambda ),\;J=\text{det}F,\;{\bar{I}}_{1}=\text{tr}\bar{B}$$ (1)

contains Neo-Hookean form with two constants *C*_1_ and *D*_1_ (Table 1) defining matrix substance and transverse part *F*_2_(*λ*) depending on the stiffness of collagen fibres [26,27,28]. The function *F*_2_(*λ*) fulfils the conditions

$$\lambda \frac{\partial F_{2}(\lambda )}{\partial \lambda }=\{\begin{array}{ll} 0, & \lambda \leq 1\;,\\ C_{3}\left(exp\left(C_{4}(\lambda -1)\right)-1\right), & 1<\lambda <\lambda^{*},\\ C_{5}\lambda +C_{6}, & \lambda \geq \lambda^{*}, \end{array}$$ (2)

where *λ* is fibre stretch, λ* is limit value of stretch corresponding to straightened fibre and *C*_3_, *C*_4_, and *C*_5_ are material constants given in Table 1. The constant *C*_6_ was calculated from continuity condition for λ*. Fibre stretch is connected with deformation gradient **F** by relation *λ***a**(**x**) = **F**(**X**)∙**a**_0_(**X**), where **a** and **a**_0_ are the orientations of fibres in the current configuration **x** and the reference configuration **X**, respectively. The material model for ligaments was implemented as a user subroutine in FEM code Abaqus 6.14 (Dassault Systemes Simulia Corp., Providence, RI, USA) [26].

Connections of cartilage surfaces and ligament attachments to bone surfaces were introduced as rigid constraints. The frictionless hard contact approach was used to describe the interaction between appropriate articular cartilage surfaces and menisci surfaces. Reduced automatic stabilisation (factor = 0.5) was used for contact to improve convergence during analyses.

### Finite element mesh

The finite element models of the knee joint were prepared in the Abaqus code. The elastic parts of models were meshed using the first order, 8-node solid elements (C3D8). Second order, 10-node tetrahedral solid elements (C3D10) were used for the ligaments and rigid shell elements for the bones. A non-uniform finite element mesh (average 1 mm size of finite element) was generated in the models. The discretisation was refined in regions with high stress values. A total of 103,453 elements and 148,238 nodes were created in model 1. The convergence analysis was performed in order to verify the generated finite element mesh.

### Boundary conditions, initial state

Since the geometry of the cadaver knee was registered in the fully extended position, the initial strains were introduced in the ligaments. For each ligament, total stretch *λ*_0_ ≈ 1.05 was imposed by modifying deformation gradient in an initial step of calculations [29]. The approximate 5% value was used due to the lack of experimental data for the analysed geometry [28].

At the tibia RP, five degrees of freedom (DOFs) were fixed, except for varus/valgus rotation ϕ_z_. A translation in the direction of mechanical axis *u*_y_, varus/valgus rotation ϕ_z_ and external/internal rotation ϕ_y_ was released at the femur RP. These free DOFs are shown schematically in Fig 1a. The translational DOFs and flexion/extension rotation were constrained because the knee was analysed in the stance position. The computations for all models were performed using static solver and taking into account nonlinear effects. The knee joint was loaded at the femur RP by compressive force of 1000 N, acting in the direction of mechanical axis.

## Results

**Fig 2:**
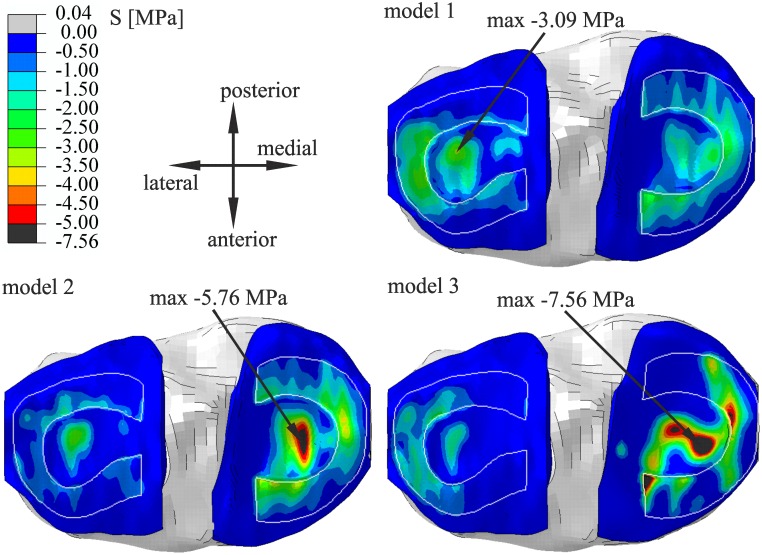
Comparison of principal compressive stresses on the tibial cartilages for model 1 of the intact knee and models with reduced thickness of articular cartilages: model 2 with congruent contact surfaces and model 3 with incongruent contact surfaces. The edges of the menisci are highlighted in white.

We compared the results for the following models: the model of intact knee (model 1) and two models of knee with reduced thickness of medial cartilages: model 2 with congruent contact surfaces and model 3 with incongruent contact surfaces. All of the values in tables and figures are reported for the final value 1000 N of the external force. The distribution of principal compressive stresses on the tibial plateau for all models is presented in Fig 2. The location and value of extreme stress are also presented in contour maps. The maximal value of compressive stress was obtained in model 3.

The values of meniscus displacement *w* in medial-lateral direction (Table 2) give information about the reduction of cartilage surface that is protected by meniscus. We observed the greatest value of meniscal external shift in the medial compartment in model 3. In the lateral compartment the values of *w* were about 0.73–1.27 mm lower than in the medial compartment. The reduction of cartilages’ thickness in the medial compartment (models 2 and 3) resulted in considerable vertical displacement *v* between the medial articular surfaces (Table 2) as well as the considerable value of the varus rotation (Table 2). The values of the relative translation between articular surfaces of bones in the lateral-medial direction are compared in Table 2. The greatest value of *u* was obtained for model with incongruent contact surfaces (model 3).

**Table 2:** Values of meniscal external shift *w*, vertical shift of the articular surfaces *v*, varus rotation and relative translation between articular surfaces *u* for analysed knee models

	Lateral compartment		Medial compartment		varus rotation [°]	*u* [mm]
	*w* [mm]	*v* [mm]	*w* [mm]	*v* [mm]		
model 1	0.028	0.834	0.755	0.994	0.22	0.132
model 2	-0.295	0.518	0.744	3.318	3.87	-0.905
model 3	0.188	0.333	1.453	3.081	3.80	-1.821

**Fig 3:**
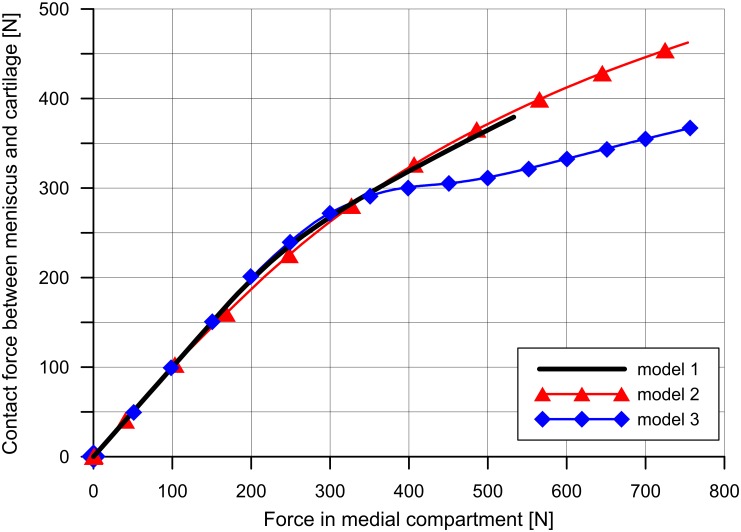
The contact force between the tibial cartilage and the medial meniscus as a function of force in the medial compartment.

Varus rotation changes the distribution of compressive force between the lateral and medial compartment (Table 3). We observed an increase in the contribution of medial compartment in load transmission from 52% in model 1 to 75% in models 2 and 3. The comparison of cartilage and menisci role in load carrying process is presented in Table 3, separately for lateral and medial compartments. An increase in the force acting between articular cartilages and the tibial cartilage and the meniscus in the medial compartment (Table 3) was observed in model 2 in comparison to model 1. In model 3, in the medial compartment, we noticed a greater value of forces acting between cartilages and lower value of forces between the cartilage and meniscus than in model 2 (Table 3). Fig 3 shows the change in contact force between tibial cartilage and medial meniscus as the function of the force in the medial compartment. In the initial phase, the curves are consistent for all models, because the medial meniscus carries the whole force in the medial compartment. After the onset of articular cartilage contact, we observed a lower increase of meniscus to cartilage contact force in model 3 than in the other models (Fig 3, force > 300 N).

**Table 3:** The contribution of menisci and articular cartilages in carrying external load.

	Force between articular cartilages		Force between tibial cartilage and meniscus				Total force	
	lateral	medial	lateral		medial		lateral	medial
	[N]	[N]	[N]	[%]*	[N]	[%]*	[N]	[N]
model 1	165.05	153.61	331.7	66.8%	379.27	71.2%	496.75	532.88
model 2	82.96	291.22	174.34	67.8%	462.53	61.4%	257.3	753.75
model 3	42.13	392.55	216.22	83.7%	367.75	48.4%	258.35	760.3

*percentage values are computed relative to total force values

**Fig 4:**
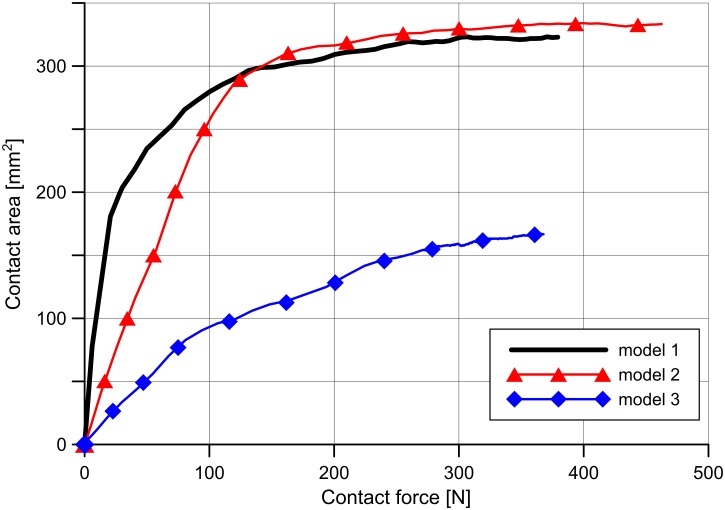
Relation between the contact area and the contact force for contact surfaces of meniscus and tibial cartilage in the medial compartment.

The values of contact areas between articular cartilages and between articular cartilage and menisci are presented in Table 4. The contact area between the tibial cartilage and the meniscus in the medial compartment is about 50% lower in model 3 than in model 2 (Table 4). We observed a decrease of contact area in model 3 also for other contact surfaces (Table 4), with the exception of the contact area between articular cartilages in the medial compartment which can be attributed to the greater contact force in model 3 (Table 3). The change of contact area between the medial meniscus and the tibial cartilage as a function of contact force is shown in Fig 4. As a measure of congruence, we propose the slope of the curves in Fig 4, since for a given value of contact force, the slope of the curve (value of the contact area) depends on the congruency between the contact surfaces.

**Table 4:** The comparison of contact area [mm^2^] for contact surfaces in analysed knee models.

	Contact area between articular cartilages [mm^2^]		Contact area between tibial cartilage and meniscus [mm^2^]		Contact area between femoral cartilage and meniscus [mm^2^]	
	lateral	medial	lateral	medial	lateral	medial
model 1	130.80	134.32	250.69	322.88	287.27	348.28
model 2	64.01	133.02	223.70	333.33	206.23	291.92
model 3	38.87	143.49	193.09	166.66	163.34	181.88

## Discussion

In order to investigate the mechanical characteristics of the meniscus in the osteoarthritic knee, we chose to use finite element analysis. This method has proven to be a very useful tool for creating complex models of an organ with advanced analysis of a connection between structure and function [30]. Since the essential step in this method is the verification of simulation results, we confirmed that our results for the intact knee model are consistent with literature data [22,31,32,33].

The increased vertical shift of the articular surfaces (Table 2) is interpreted as a narrowing of joint space in clinical tests [34]. Laxafoss et al. described changes in the angle of knee joint in OA and asymmetric narrowing of the joint space [35]. They documented the tendency for the axis of the OA knee to depart in the direction of the diseased compartment in a linear relationship to the severity of the osteoarthritis. This causes the force vector to pass more medially. We observed, as have other authors, that the increase of the total force in the medial compartment (Table 3) is connected to the varus angle (Table 2) [36]. Comparing our two models 2 and 3 we noticed that the total forces in the medial compartment increased to a similar value (Table 3).

Under physiologic conditions, the medial meniscus carries between 40% and 80% of the load in the medial compartment of healthy knee [3]. The way the meniscus contributes to the force transmission in varus OA knee and cartilage degeneration remains unclear. Arno et al. showed that with cartilage loss, the intact meniscus loses its ability to distribute stress and thus is unable to prevent degeneration in medial knee OA [11]. Whether the functional meniscus in OA knee can lose its protective role is debatable [11,12]. In our study we made comparisons with the use of two models differing in the congruence of articular surfaces. We showed that the contribution of medial meniscus to the force transmission in model 2 (61.4%) is greater than in model 3 (48.4%) (Table 3). This is related to the contact area between the tibial cartilage and the meniscus in model 3 being 50% lower than in model 2 (Table 4). Fig 4 confirms that the reason for the smaller contact area in model 3 is the deteriorated congruency between contact surfaces. Another factor reducing the contact area between the meniscus and the cartilage is the meniscal external shift. Its value showed a twofold increase in the model with incongruent contact surfaces in comparison to the model with congruent contact surfaces (Table 2). The consequence of smaller contact areas is lower values of contact force between the medial meniscus and the tibial cartilage in model 3 than in model 2 (Fig 3). This leads to the substantial decrease in the contribution of the medial meniscus in load transmission with the increase of compressive load on the cartilage. According to the previous research, cartilage degeneration is not a geometrically regular process [11,37,38]. Model 2 was designed in order to confirm the influence of congruence of articular surfaces on the occurrence of the external meniscus shift and the contribution of medial meniscus. The significance of the congruence of contact surfaces on the contribution of the menisci in load transmission is an important argument for preservation of the meniscus. Researchers are still working on new methods of menisci repair to improve the effect of surgery [39,40].

In model 3 we also demonstrated that the lack of congruence causes relative translation *u* between the articular surfaces (Table 2). This additionally contributes to the reduction in contact area between the meniscus and the cartilage. We noticed that this phenomenon also affects the strain of ligaments, which will be the object of further research. The value of the contact area between the medial meniscus and the tibial cartilage in models 1 and 2 is stabilising for contact forces greater than 150 N (Fig 4), as it is close to the total area of meniscus contact surface. In model 2, the initial congruency is worse than in the model 1 (Fig 4), due to the relative translation between articular surfaces of bones and greater stiffness of articular cartilage in model 2 due to their reduced thickness.

Comparing the factors altering the maximal compressive stresses between cartilages among all of the models, we showed that the most influential is the change in knee alignment toward genu varum and a reduction of the contact area between cartilages. Varus malalignment contributes to the substantial increase of the total force in the medial compartment (Table 3). The minimum principal compressive stress was obtained in model 3 (Fig 2) due to the smaller role of the medial meniscus in load transmission and the non-uniform distribution of stresses on cartilage surfaces.

In a previous study, Wenger et al. evaluated the position, shape and size of the menisci in subjects with knee osteoarthritis with the use of magnetic resonance imaging [12]. Their findings indicate an altered lateral meniscal position in medial knee osteoarthritis. They speculated that varus malalignment could lead to the unloading of the lateral compartment and meniscus hypertrophy with bulging. In our study, model 3 confirmed their hypothesis. In the lateral compartment, we observed a decrease in forces acting between cartilages and the lateral meniscus (Table 3) due to varus malalignment. In addition, we observed that changes of meniscal position could be related to the distribution of compressive stresses between the cartilage and the meniscus (Fig 2). In model 3, the load is borne mainly via the centre part of the lateral meniscus, which increases the force acting on the meniscus in the lateral direction. Thus, we noticed external meniscus shift only in model 3. The change in stress distribution could be related to the relative translation between articular surfaces. The small values of external shift of the lateral meniscus (Table 2) are connected with its flattened geometry in the centre part (small slope angle) [22].

Our study has certain limitations. First, there is a lack of physical validation for the knee joint geometry from the Open Knee project. Since the paper is not clinical in nature, we decided that literature-based validation would be sufficient [41]. Another limitation is the assumption of the same material properties for cartilages and menisci in the intact knee model and the knee models with medial compartment osteoarthritis. Moreover, we used linear, elastic and isotropic model material for articular cartilages and transversely isotropic model for other soft tissues, since only time-independent analyses were performed. However, the assumed simplified homogenous model of the cartilage is sufficient to perform this study, because the articular cartilage stiffness has little impact on meniscus biomechanics [42,43]. Only static analyses for one compressive load case were performed. Since small velocities are expected, and local dynamic effects are directly negligible [44]. The possible error in calculation of the mechanical axis and position of bone RPs could slightly change the value of varus rotation and the consequent distribution of force between the lateral and medial compartments. However, it has a very small impact on the main results of the study.

## Conclusions

In conclusion, we demonstrated that the change in cartilage geometry in knee osteoarthritis may meaningfully affect the biomechanics of the intact meniscus. We proved that surface incongruence between the meniscus and the cartilage considerably reduces the contact area between them and thus the stress distribution capacity of the meniscus. Moreover, we demonstrated a relation between changes in cartilage geometry and the risk of meniscus extrusion in both knee joint compartments. The above conclusions contributes to a better understanding of the phenomena described in clinical studies, especially in the case of the interaction between various tissues in the knee joint.
